# Exercise-Based Renal Rehabilitation: A Bibliometric Analysis From 1969 to 2021

**DOI:** 10.3389/fmed.2022.842919

**Published:** 2022-03-21

**Authors:** Fan Zhang, Jing Ye, Yan Bai, Hui Wang, Weiqiong Wang

**Affiliations:** ^1^Department of Nephrology, Longhua Hospital, Shanghai University of Traditional Chinese Medicine, Shanghai, China; ^2^Blood Purification Center, Longhua Hospital, Shanghai University of Traditional Chinese Medicine, Shanghai, China; ^3^Department of Cardiology, Longhua Hospital, Shanghai University of Traditional Chinese Medicine, Shanghai, China; ^4^Department of Anorectal, Longhua Hospital, Shanghai University of Traditional Chinese Medicine, Shanghai, China

**Keywords:** exercise-based renal rehabilitation, bibliometric analysis, chronic kidney disease, VOSview, Citespace

## Abstract

Chronic kidney disease (CKD) is a growing global health challenge with an increasing incidence rate. Exercise-based renal rehabilitation is an evidence-based, multidisciplinary, and comprehensive intervention designed to improve the physical and psychological condition of patients with CKD. The knowledge structure, research hotspots, and development trends in exercise-based renal rehabilitation have not been systematically described. The aim of this study was to provide a bibliometric perspective of the progress in this field. Publications about exercise-based renal rehabilitation were retrieved from the Web of Science Core Collection, using the terms “exercise,” “physical activity,” and “chronic kidney disease.” Annual publications, subject categories, countries, authors, references, and keywords in this field were visually analyzed using the Citespace, VOSview, and Excel software. A total of 4,610 publications were analyzed, with a steady increase in publications in the field. Overall, the United States is the major contributor to the study of exercise-based renal rehabilitation. Johansen KL and Painter P are the key researchers in this field. Keyword analysis shows that research hotspots in this field include exercise/physical activity for different stages of CKD, exercise-based renal rehabilitation for frailty, and physical activity management for CKD. These findings will make understanding exercise-based renal rehabilitation research better and inform about future research ideas.

## Introduction

Chronic kidney disease (CKD) is a progressive disorder, which can lead to end-stage renal disease (ESRD), and requires renal replacement therapy, including dialysis (i.e., hemodialysis or peritoneal dialysis) or kidney transplant (1). Patients at different stages of CKD experience substantial burdens, including cardiovascular injury (2), exercise intolerance (3), physical/psychological fatigue (4), and poor health-related quality of life (5).

Based on current evidence, the guidelines for managing CKD by the Japanese Society of Renal Rehabilitation (JSRR) identify renal rehabilitation as an effective and safe intervention (6). Renal rehabilitation is defined as “a long-term comprehensive program that includes exercise therapy, diet and water management, pharmacotherapy, education, and psychological/spiritual support to reduce the physical/mental effects of kidney-based disease and dialysis” (7). Exercise therapy is a cornerstone of renal rehabilitation (8). It may alleviate symptoms and improve function and exercise tolerance in patients with CKD and, therefore, may be a promising intervention for such a population (9). However, the issue of physical inactivity causing the decreased quality of life (10) and disease progression in patients with CKD (11) remains underappreciated. In November 2016, an international group of researchers and clinicians met in Chicago as the Global Renal Exercise (GREX) Working Group to discuss research priorities related to exercise in CKD (12). They meet regularly to foster collaborative interdisciplinary research and innovation to develop practical and feasible strategies to increase physical activity in patients with CKD. At present, many research groups worldwide are working on exercise-based renal rehabilitation.

Unlike a systematic and scoping review, a bibliometric analysis refers to the qualitative and quantitative evaluation of a specific research field using mathematical and statistical methods to understand the knowledge structure and explore development trends (13). The method allows the comparison of contributions and collaborations across subject categories, authors, countries, and journals. Furthermore, the bibliometric analysis can predict the hotspots and trends within a specific research area through information visualization (13, 14), which are also important and of interest in the medical field. In recent years, bibliometric studies have been widely used in public health and/or clinical research (15–17). However, there is still a lack of analysis for renal rehabilitation.

This study performed a comprehensive bibliometric analysis of exercise-based renal rehabilitation research publications from 1969 to 2021, focusing on annual publications, subject categories, countries, authors, references, and keywords. The results will provide new perspectives and references for future renal rehabilitation research.

## Methods

### Data Sources

Data were extracted from the Web of Science Core Collection (Science Citation Index Expanded: 1945). The database covers over 21,000 peer-reviewed, high-quality academic journals, including open-access journals published in over 250 medical, social science, and humanities disciplines worldwide, and is widely used for bibliometric analysis. Moreover, the database provides access to the authors, country, affiliation, keywords, and references cited for each publication, which is necessary for this study.

### Search Strategy

**Figure 1:**
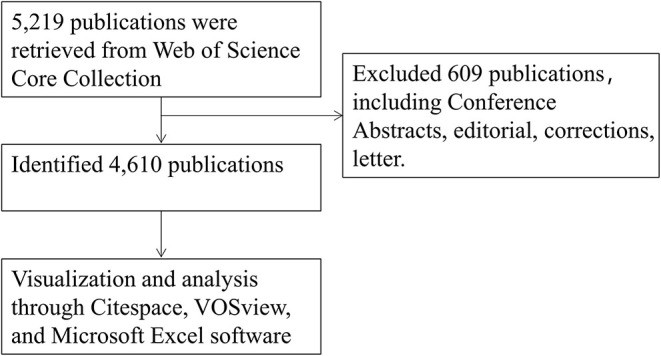
A flowchart of the publication screening process.

The searched terms were “exercise,” “physical activity,” “chronic kidney disease,” “peritoneal dialysis,” and “hemodialysis” (the detailed retrieval strategy was shown in Supplementary File 1). The search language was not restricted, and the retrieval time spanned from inception to December 9, 2021. The publication type was restricted to reviews and articles, with 4,610 publications included eventually (Figure 1).

### Data Analysis

All downloaded documents were imported to the VOSviewer (version 1.6.15) software, Citespace (version 5.8.R2) software, and Microsoft Excel 2019.

VOSviewer and CiteSpace are software for building and visualizing bibliometric networks, including countries, journals, and authors based on citation, co-citation, or coauthorship. Furthermore, it can be used to visualize co-occurrence keywords to understand the knowledge structure of a research field and to explore trends (18, 19). Where the nodes represent research items, the larger the size, the more frequently the entries or citations appear. The links between nodes describe the co-occurrence or co-citation between these nodes, and the thickness indicates the strength of the correlation: a thicker line indicates a stronger connection. The shade or hue of node and connection colors indicates the chronological order of the item occurrences (20).

Centrality was used to assess the importance of each node in the network. Nodes with centrality >0.1 were shown as purple circles. The thickness of the purple circles increased with the degree of centrality, a metric associated with the translational potential of scientific contributions (20).

The main procedures for setting up a bibliometric analysis using Citespace software were as follows: (1) importing the literature and adjusting the data format, (2) adjusting the time slice (1 year), (3) restricting the term source (i.e., subject, country, and keywords), and (4) setting the selection criteria on each slice (i.e., the top 50 cited or occurring items in each entry).

Using VOSviewer software, the main procedures were then (1) importing the literature and formatting the data, (2) restricting the term sources (i.e., authors, citations, and keywords), and (3) selecting the minimum number of co-occurrences and co-citations (i.e., threshold).

## Results

### The Trends in Global Publication

**Figure 2:**
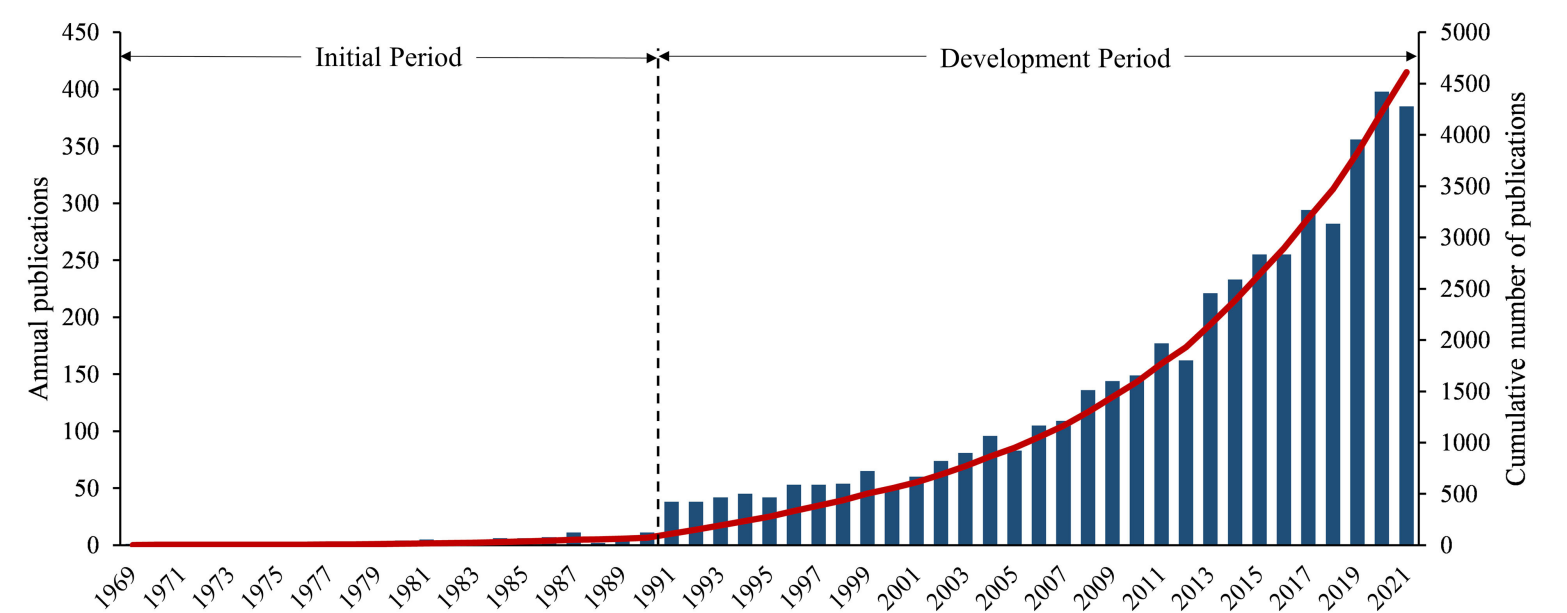
Annual number of publications in this field.

A total of 4,610 publications related to exercise-based renal rehabilitation from 1969 to 2021 were retrieved from the database. Based on the number of annual publications, this period was preliminarily divided into two phases (Figure 2), namely, the first phase was considered the initial period (1969–1990), with an average of three publications per year, and the second phase, from 1991 to present, was considered as the development period, with an average of 146 publications per year.

### Analysis of Subject Categories

**Figure 3:**
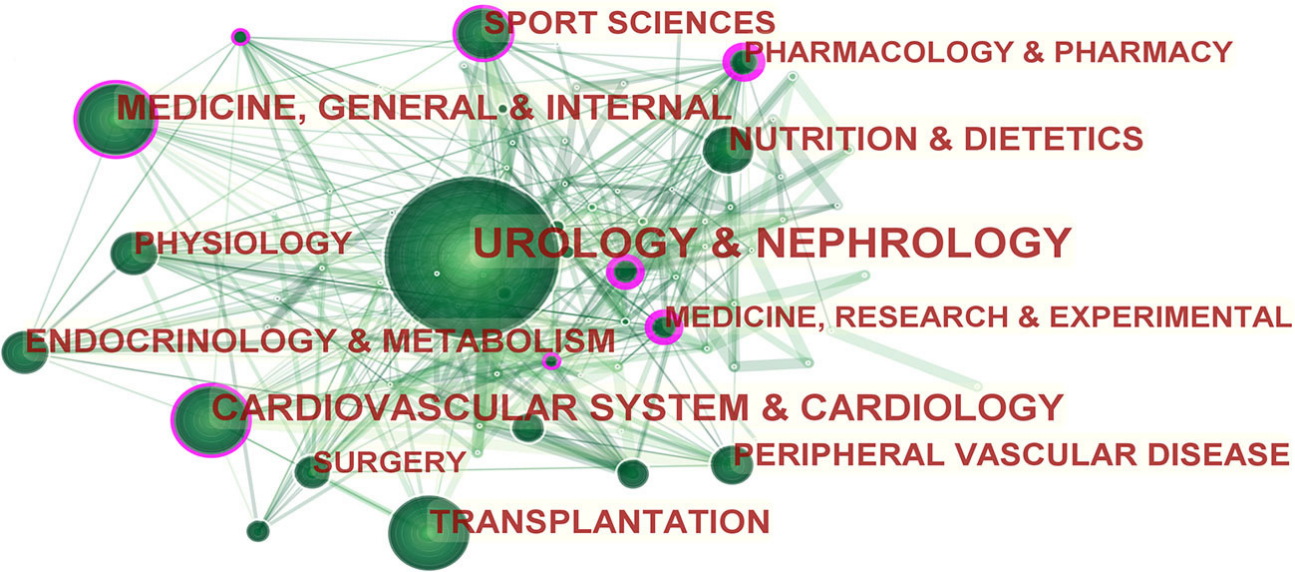
Subject category co-occurrence of publications about exercise-based renal rehabilitation. Circled nodes represent output subjects; connections between nodes indicate cooperative relationships.

The co-occurrence analysis of subject categories allows us to intuitively understand the main subjects involved in a research field. In Figure 3, the purple circle on the edge of the node circle indicates that this node has a high intermediate centrality value. In addition to nephrology, exercise-based renal rehabilitation research is mainly centered on the cardiovascular system, internal medicine, and organ transplantation. Furthermore, we listed the top 10 subject categories with centrality in Table 1. It shows that research among exercise-based renal rehabilitation involves multiple disciplines and fields.

**Table 1:** Top 10 subject categories based on centrality related to exercise-based renal rehabilitation.

**Rank**	**Subject categories**	**Centrality**	**Frequency**
1	Pharmacology/Pharmacy	0.30	155
2	Public, environmental & occupational health	0.23	128
3	Medicine, research & experimental	0.21	171
4	Cardiovascular system & cardiology	0.17	547
5	Sport medicine	0.14	231
6	Medicine & general internal	0.12	454
7	Biochemistry & molecular biology	0.12	78
8	Physiology	0.10	211
9	Pediatric	0.10	78
10	Immunology	0.08	88

*The higher the centrality, the more influential the node is in the network structure mapping. Chen et al. considered that a centrality >0.1 indicates that the node is more important and plays a bridge role in the mapping (20)*.

### Distribution of Countries/Regions

**Figure 4:**
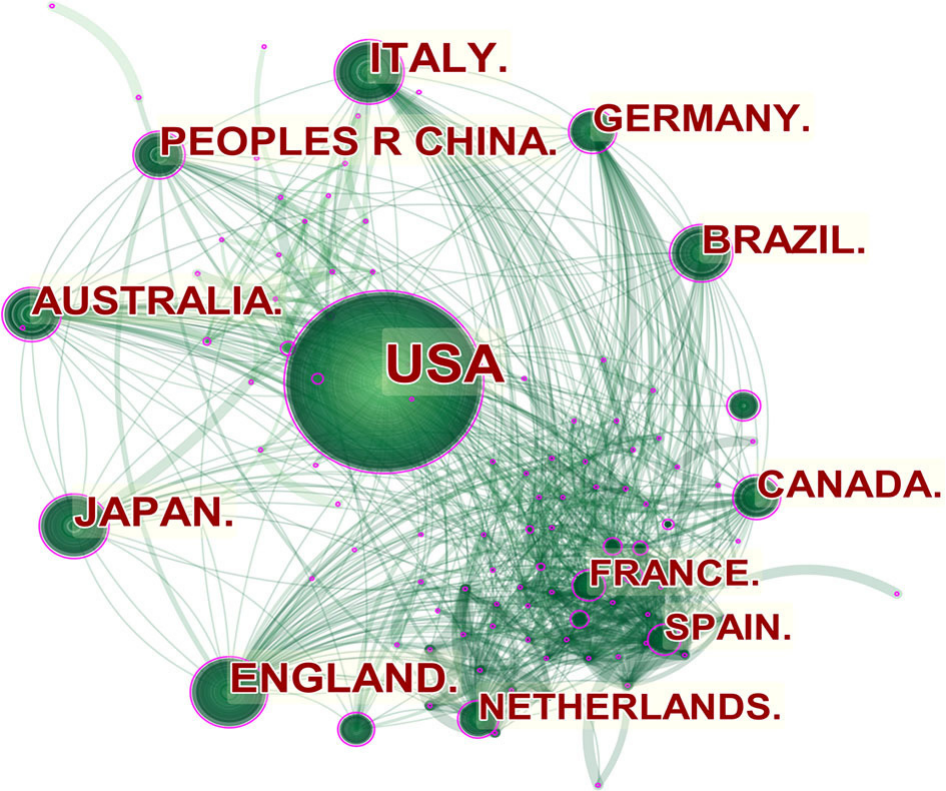
Countries' network of publications about exercise-based renal rehabilitation. Circled nodes represent output countries; connections between nodes indicate cooperative relationships.

A total of 106 countries/regions contributed to publications in exercise-based renal rehabilitation. A visual analysis of the country distribution shows that countries such as the United States, England, Japan, and Italy are the most notable countries for coauthorship publications, and these countries not only have the highest number of publications but also rank highest in centrality (Figure 4, Table 2). From the citation perspective, studies from the United States had the highest number of citations (i.e., 65,124 citations), followed by those from England (8,179 citations), Australia (6,496 citations), Canada (5,920 citations), and Japan (5,666 citations) (Table 2).

**Table 2:** Top 10 countries/regions based on publications related to exercise-based renal rehabilitation.

**Rank**	**Country/region**	**Publications**	**Centrality**	**Citations**
1	USA	1,611	0.96	65,124
2	England	411	0.13	8,179
3	Japan	366	0.02	5,666
4	Italy	322	0.10	5,481
5	Australia	258	0.03	6,496
6	China	242	0.00	4,335
7	Germany	240	0.08	4,629
8	Brazil	240	0.00	2,649
9	Canada	206	0.01	5,920
10	France	186	0.11	2,316

### Analysis of Authors

**Figure 5:**
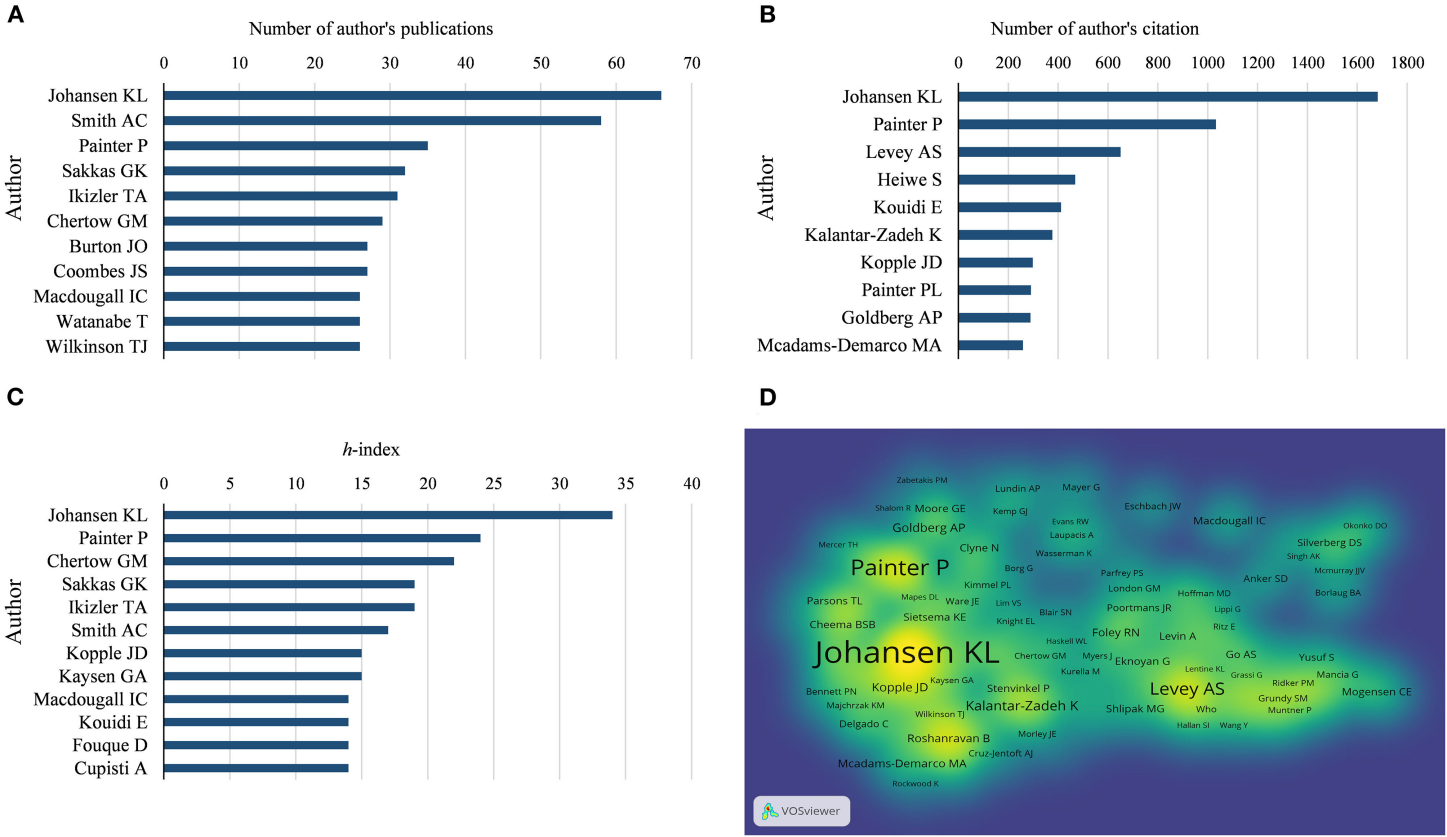
Analysis of authors. **(A)** The number of author's publications. **(B)** The number of author's citations. **(C)** Authors' overall *h*-index. **(D)** Density visualization of co-citation of authors. The *h*-index is a common metric for measuring the impact of researchers, which measures the number of articles with at least h citations per article by the author (21).

In terms of the number of publications in the field of exercise-based renal rehabilitation, Johansen KL was the most productive author, with 66 publications, followed by Smith AC (58 publications), Painter P (35 publications), Sakkas GK (32 publications), and Ikizler TA (31 publications) (Figure 5A). Concerning author impact factor, publications from Johansen KL had the highest *h*-index (34), followed by Painter P (24), Chertow GM (22), Aakkas GK (19), and Ikizler TA (19) (Figure 5B).

In terms of co-citations, a total of 317 authors with more than 50 citations in the field were analyzed. Johansen KL (1,682 citations) had the highest number of citations, followed by Painter P (1,033 citations), Levey AS (651 citations), Heiwe S (469 citations), and Kouidi E (412 citations) (Figures 5C,D).

### Co-citation Analysis

**Figure 6:**
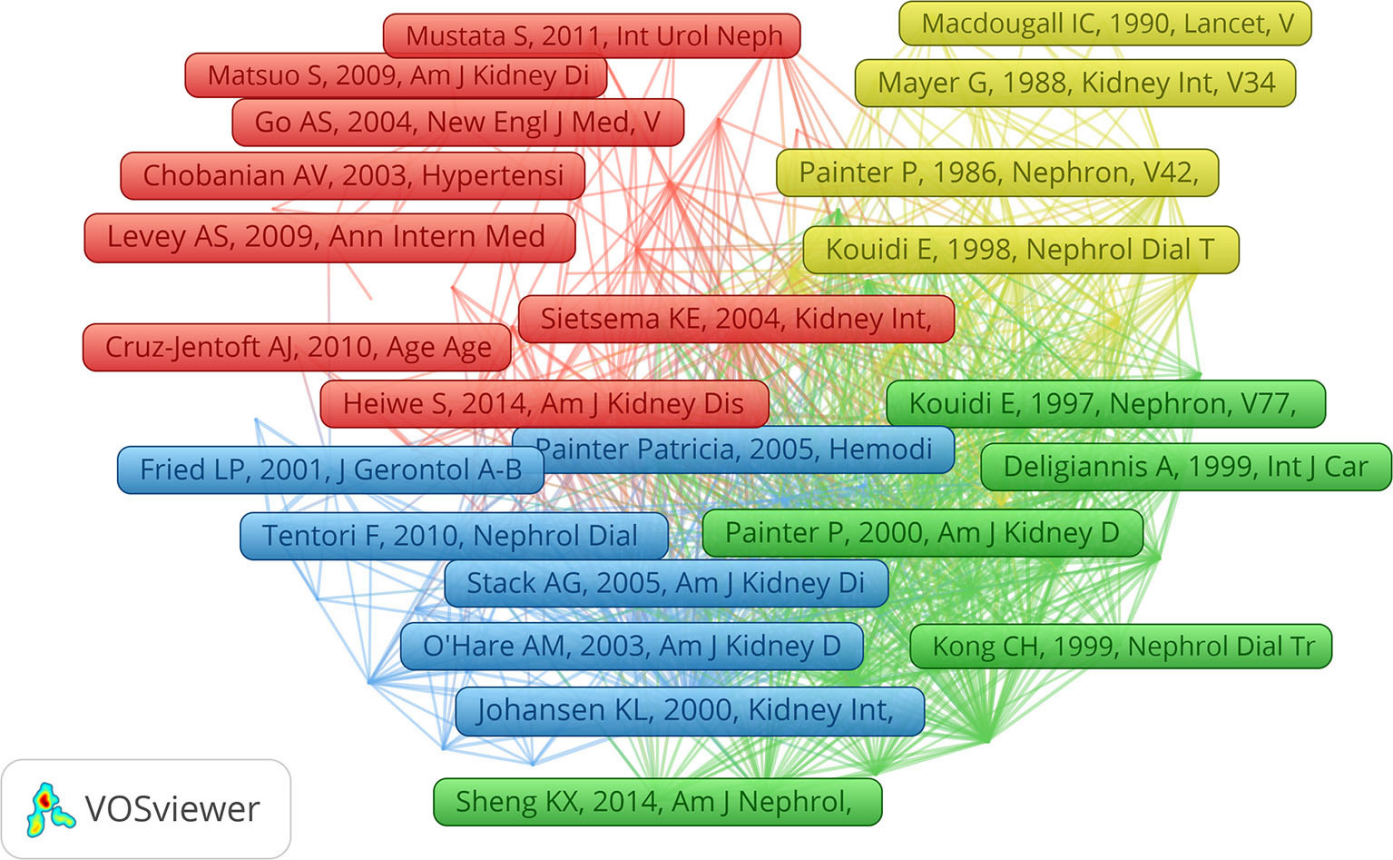
Network map of co-citation of references with more than 50 citations.

There were 121 references that were co-cited with more than 50 citations (Figure 6). The five most cited references were published by Johansen KL (232), O'Hare AM (174), Kouidi E (167), Heiwe S (166), and Painter P (165) (Supplementary File 2). Furthermore, based on the references with the strongest citation bursts, Table 3 shows the top 10 co-cited references. Classic citations in the field of exercise-based renal rehabilitation have been frequently cited over the past decade, such as physical function in patients with CKD, especially dialysis-dependent patients, optimal exercise prescription for patients with CKD, and nutritional strategies to optimize exercise outcomes, implying that this field of research will continue to be explored in the future.

**Table 3:** Top ten references with the strongest citation bursts.

**References**	**Year**	**Strength**	**Begin**	**End**	**1969–2021**	**Conclusion**
Johansen et al. (22)	2003	19.45	2005	2008	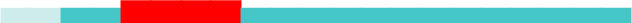	HD patients were weaker, less active, and walked more slowly than controls.
Johansen et al. (23)	2006	22.4	2007	2011	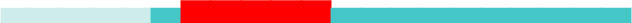	Nandrolone decanoate and resistance exercise produced anabolic effects among HD patients, resulting in improved muscle tissue
Johansen et al. (24)	2007	23.83	2008	2012	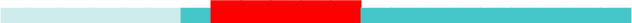	This article reviews the current status of research on exercise in ESRD patients.
Heiwe et al. (8)	2011	26.54	2012	2016	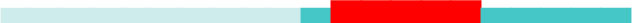	Exercise benefits physical fitness, walking capacity, cardiovascular parameters, quality of life, and nutritional parameters in CKD patients.
Johansen et al. (25)	2012	27.35	2013	2017	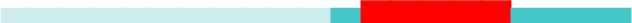	This review covers the rationale for exercise in non-dialysis patients with CKD and the effects of exercise training on physical functioning, progression of kidney disease, and cardiovascular risk factors.
Roshanravan et al. (26)	2013	26.04	2014	2018	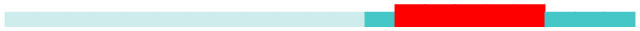	Impaired physical performance of the lower extremities is common in CKD and strongly associated with all-cause mortality.
Heiwe et al. (9)	2014	41.15	2015	2019	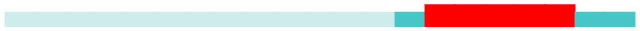	Regular exercise training generally is associated with improved health outcomes in individuals with CKD.
Sheng et al. (27)	2014	20.74	2016	2019	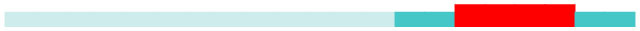	Intradialytic exercise can improve Kt/V, VO_2peak_, and the physical quality of life, and intradialytic exercise is safe for HD patients.
Barcellos et al. (28)	2015	19.25	2016	2021	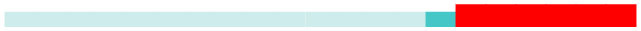	The benefits of exercise in dialysis patients are well established, but the best exercise protocol for CKD patients is also still unclear.
Manfredini et al. (29)	2017	30.79	2018	2021	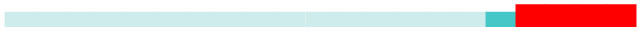	A simple, personalized, home-based, low-intensity exercise program may improve physical performance and quality of life in dialysis patients.

*CKD, chronic kidney disease; HD, hemodialysis; VO_2peak_, peak oxygen uptake*.

### Analysis of Keywords

**Figure 7:**
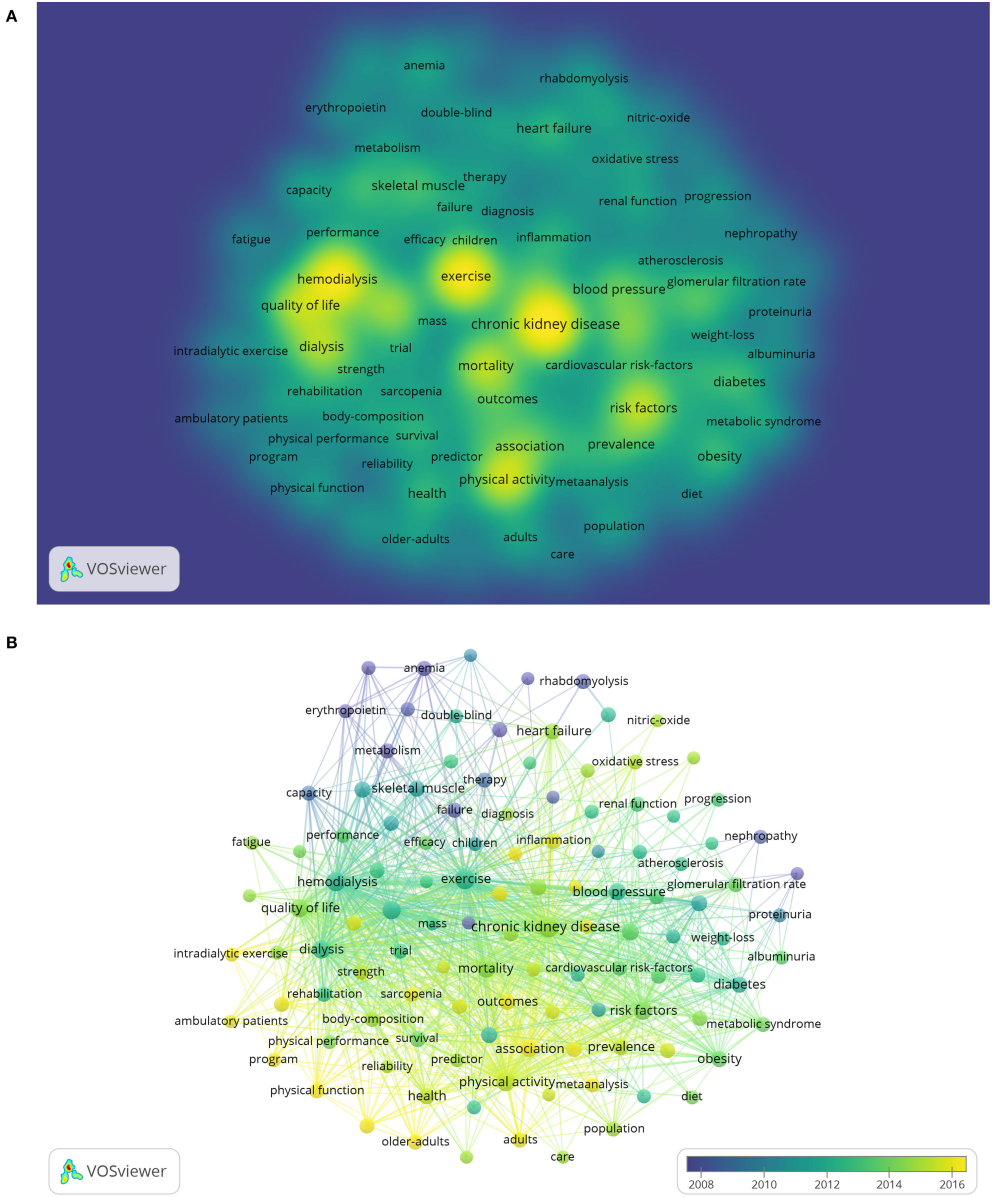
Co-occurrence analysis of keywords. **(A)** Overlay visualization. **(B)** Density visualization.

This study analyzed 118 keywords that appeared more than 50 times (Figure 7A). The colors in the overlay visualization shown in Figure 7B represent the average publication year of the identified keywords. Most keywords were published after 2012, with greener or yellower colors. As shown in the figure, the research keywords in the field of exercise-based renal rehabilitation included different stages of CKD, exercise/physical activity, physical activity and mortality, CKD and frailty/sarcopenia, intradialytic exercise, and adult and elderly.

## Discussion

### General Information

This study described the landscape of exercise-based renal rehabilitation by analyzing the subject categories and the contribution of countries, journals, and authors. The trend of annual publications published from 1969 to 2021 demonstrated a stable growth.

From a macroscopic perspective, exercise-based renal rehabilitation is a typical multidisciplinary collaboration for the comprehensive management of patients with CKD, as confirmed by the disciplinary co-occurrence analysis. The subject co-occurrence map demonstrates the connectedness between disciplines surrounding the field of exercise-based renal rehabilitation. As shown in Table 1, pharmacology/pharmacy and public, environmental, and occupational health ranked the top two in centrality; not surprisingly, the kidney is an essential organ for drug metabolism and excretion, and pharmacokinetics can change significantly in patients with CKD. Therefore, it is vital to fully understand the pharmacokinetic characteristics of patients with CKD to reasonably select the dose of drugs and give individualized management. In addition, the high centrality of cardiology, sports medicine, and experimental science indicates that exercise-based renal rehabilitation must assess the systemic status, comorbidities (especially, cardiovascular disease), the nutritional status, and exercise capacity of patients with CKD to propose an individualized exercise program. For this purpose, it is necessary to establish a team of cardiologists, physiotherapists, exercise specialists, and physiologists to promote an exercise culture to improve the physical activity level of patients with CKD and to promote prognosis (30).

A quantitative and visual analysis of the distribution of countries/regions shows that the United States and England are the leading countries in the field of exercise-based renal rehabilitation research. As shown in Figure 3, there is a greater density and breadth of collaboration between the various countries, especially from the European and American regions. Unfortunately, we rarely see research participation from Asian countries, and this situation may hinder the development of this field to some extent. Therefore, we suggested that research institutions from Europe and the United States strengthen cooperation and communication with Asian countries to promote renal rehabilitation research.

From the perspectives of author contributions and co-cited authors, Johansen KL, an American researcher from the University of California, contributed 66 publications. The other representative was Painter P. It is noteworthy that both researchers are ranked at the top in terms of *h*-index and citations. Johansen KL found significant muscle atrophy in hemodialysis-dependent patients with CKD based on the MRI and also found that the severity of muscle atrophy increases gradually with the progression of the disease (31, 32). Significant atrophy was associated with poor physical performance, as evidenced by greater weakness, inactivity, and slower walking than healthy sedentary controls (22). Therefore, the authors suggested that physical activity should be increased to address the comorbidity of muscle atrophy (22). Subsequently, Johansen KL conducted a series of high-quality trials on exercise for patients with CKD; these studies provided moderately reliable evidence that exercise interventions can increase aerobic capacity and muscle function (23), can improve cardiovascular health (33), and can reduce all-cause mortality (34) in patients with CKD.

Painter P, from the University of Utah, is a facilitator in renal rehabilitation. Painter P has focused on physical function testing and exercise training in patients with CKD. Her research reported low physical function in patients with CKD, and she promotes exercise interventions in patients with CKD (35). She coauthored the expert consensus of the British Association for Sports and Exercise Science on exercise therapy for patients with CKD, providing recommendations for effective exercise therapy clinical practice (36). In addition, Painter P contributed to the formation of the “Exercise in CKD Working Group,” which specifies two major missions, namely, (1) to identify specific areas of research needed to strengthen data on the efficacy of exercise and (2) to establish a clearinghouse of resources related to exercise in CKD and to provide guidance to clinicians on their use (37).

### The Hotspot and Frontiers

The keywords can reveal the core content and research theme of the publication. We could understand the current research focus and development trends of a particular field through keyword co-occurrence and reference co-citation analysis. According to the overlay visualization and density visualization and strongest citation bursts, we determined the research hotspot and development frontiers in exercise-based renal rehabilitation as described in the following sections.

### The Significance of Exercise-Based Renal Rehabilitation on the Risk of Mortality in Patients With CKD

Given the compelling relationship between physical inactivity and adverse outcomes in patients with CKD, current research in this field focuses on exercise/physical activity and the risk of mortality in such a population (as shown in Figure 7A). Physical inactivity is crucial in the high symptom burden and progressive physical dysfunction experienced by patients with CKD (38). A lack of physical activity in patients with CKD leads to anabolic disorders, resulting in decreased neuromuscular function, muscle mass and strength, and cardiopulmonary function. The inherent comorbidities of kidney disease (e.g., uremia, inflammation, and malnutrition) further aggravate the decline in functional status. In addition, patients with CKD often suffer from metabolic acidosis, which is prone to fatigue and leads to functional limitations, reduced quality of life, and further leading to decreased physical activity levels, creating a vicious circle (39). The results of the Dialysis Outcomes and Practice Pattern Study (DOPPS) showed that patients who exercised regularly had a significantly lower risk of death compared with those who did not exercise (adjusted risk ratio = 0.6, 95% confidence interval (CI): 0.47–0.77) (40). Thus, a sedentary lifestyle is a risk factor for all-cause mortality, and prescribing physical activity may be a treatment strategy for patients with CKD with significant social benefits. However, randomized controlled trials (RCTs) regarding hard endpoints such as death and cardiovascular events have mainly focused on patients of hemodialysis, while there is a lack of attention to nondialysis patients with CKD. A recently published retrospective analysis showed that those who did not complete renal rehabilitation had a 1.6-fold [hazard ratio = 1.6; 95%CI: 1.00–2.58] higher risk of combined events (i.e., mortality and cardiovascular events) compared with patients with CKD who completed, with exercise capacity measured by the incremental shuttle walk test being a significant independent risk factor (41). Further analysis found that those with improved exercise capacity had a 40% (hazard ratio = 0.6; 95%CI: 0.36–0.98) lower independent risk of comorbid events (41). This study provides encouraging evidence that exercise-based renal rehabilitation is beneficial for patients with CKD and may address their priority of maintaining independence, preventing cardiovascular events and death (35). As Hoshino stated, renal rehabilitation is a relatively new concept. Based on the urgent needs of an aging society, especially in developed countries, it is necessary to combine multidisciplinary knowledge and gather new evidence to create a sustainable environment for renal rehabilitation (7).

### The Best Nutritional Strategies for Optimizing Exercise to Improve Frailty Outcomes in Patients With CKD

Frailty is a state of increased susceptibility to physical stressors such as illness or trauma, with a prevalence of more than 60% in dialysis-dependent patients with CKD (42). Considering the impact of frailty on poor prognosis in patients with CKD, it is critical to establish operational strategies to prevent and/or reverse frailty. Although studies have confirmed the beneficial effects of exercise on frailty, it has been suggested that the physiopathological pathways underlying the development of frailty in patients with CKD are related to an imbalance in individual protein degradation relative to protein synthesis (43), as well as inadequate or inappropriate substrate utilization, and that exercise intervention alone may not sustain long-term benefits (44). Therefore, the new therapeutic approach of oral (or parenteral) nutritional supplementation combined with exercise has attracted considerable interest from nephrologists, as it is beneficial for several health indicators in the older population (45). Several studies have examined the effects of exercise combined with oral nutritional supplementation (ONS) in dialysis-dependent patients with CKD, but the results were not consistent. For example, Martin-Alemañy et al. evaluated the effects of ONS and resistance exercise on markers of nutritional status in patients of hemodialysis. Although ONS improved the nutritional status, no anabolic effect of resistance exercise on nutritional status was observed to enhance ONS (46). In another RCT, intradialytic protein supplementation and endurance exercise training over a 12-month period similarly did not observe effective changes (47). Notably, recently, the AVANTE-HEMO study presented a greater effect on physical function after 12 weeks of ONS and exercise training than ONS alone (48). Despite the negative results, interventions based on nutritional supplements and exercise training have important clinical research implications for managing frailty in patients with CKD. While exercise benefits frailty, we should recognize that dietary interventions may have immeasurable indirect beneficial effect if combined with exercise as part of a healthy lifestyle.

### Enhancing the Management of Exercise/Physical Activity in Patients With Different Stages of CKD

Despite growing evidence of the benefits of exercise for patients with CKD, the majority are not physically active enough. As renal function declines, it drops to a nadir among hemodialysis. Results of a survey of the whole spectrum of CKD showed that physical activity was more active in patients with stages 1–2 (34%), decreasing progressively from stages 3–4 and 5, and was lowest in patients of dialysis (peritoneal dialysis: 8%; hemodialysis: 6%) (49). From the perspective of healthcare professionals, the most common perceived barriers were a lack of professional guidance and advice from rehabilitation therapists (93.1%) and a lack of exercise rehabilitation knowledge (86.2%) (50). In this context, the British Renal Association (51) and the International Society for Peritoneal Dialysis (52) have published guidelines related to exercise/physical activity for patients with CKD and patients of peritoneal dialysis, respectively. From previous experience, intradialytic exercise has modified patient adherence to some extent (53), whereas patients of nondialysis or peritoneal dialysis tend to exercise in an unsupervised home/community setting. Although this mode is equally effective and safe, adherence is low. Higher levels of adherence produce better outcomes; similarly, physiological improvements may be diminished or may not occur if the exercise regimen is not followed. To date, promoting a “physically active lifestyle” for patients with CKD remains a challenge. Traditional exercise programs provide a valuable opportunity for such populations to improve physical fitness and other health conditions. However, a focus on supporting a broader range of physical activity may better meet the needs of this sedentary population, especially in older patients. It has been proposed that physical activity should be integrated into daily life rather than adding complexity to treatment regimens (54). In addition, support for daily physical activity may improve autonomy and facilitate coping with survival distress. Therefore, as we focused on renal rehabilitation, a range of actionable strategies is needed to enhance exercise/physical activity management in patients with CKD.

### Strengths and Limitations

One of the strengths of this study is that a comprehensive analysis was conducted using the Web of Science Core Collection database as the literature source, and quantitative and qualitative analyses of categories and countries were conducted using the Citespace software. Based on this, the VOSview software was used to construct a visual literature network for co-occurrence and co-citation. In addition, the visualization of the top ten references with the strongest citation bursts has undoubtedly laid the foundation for researchers to quickly understand the current status of research, hot issues, and development trends in this field.

Nevertheless, limitations are unavoidable. First, we only chose to focus on publications from solely the database. Combining the results with those from other databases (e.g., PubMed and Scopus) would have been better. However, it is worth noting that Web of Science is the most commonly used database in scientometrics, and most bibliometric software can identify information from this database. Second, as renal rehabilitation has been a developing research field in recent years, we may have underestimated recent studies published in high-quality journals, which had a low citation frequency during this period. Third, the bibliometric analysis is only an auxiliary tool, and the results may differ from real-world research conditions. Fourth, to better visualize keywords, we only analyzed the keywords appearing more than 50 times, which may ignore some recent words. Fifth, the fewer years that earlier researchers have contributed to the field compared with Johansen or Painter put them at a disadvantage. Sixth, the study period covered a long period where there was less literature, which may have introduced a risk of bias.

## Conclusion

Our findings suggest that the United States is a major contributor to the field of exercise-based renal rehabilitation. Johansen KL and Painter P are essential researchers and have significantly impacted renal rehabilitation research. Keyword analysis shows that research hotspots in this field include exercise/physical activity for different stages of CKD, exercise-based renal rehabilitation for frailty, and physical activity management for CKD.

## Data Availability Statement

The original contributions presented in the study are included in the article/Supplementary Material, further inquiries can be directed to the corresponding author.

## Author Contributions

JY and HW: conceptualization. FZ: methodology and writing–original draft preparation. FZ and YB: software and data curation. WW: writing–review and editing. All authors have read and agreed to the published version of the manuscript. All authors contributed to the article and approved the submitted version.

## Funding

This study was supported by the Second Batch of Specialist Nurse Education Program of Longhua Hospital (RC-2018-02-04).

## Conflict of Interest

The authors declare that the research was conducted in the absence of any commercial or financial relationships that could be construed as a potential conflict of interest.

## Publisher's Note

All claims expressed in this article are solely those of the authors and do not necessarily represent those of their affiliated organizations, or those of the publisher, the editors and the reviewers. Any product that may be evaluated in this article, or claim that may be made by its manufacturer, is not guaranteed or endorsed by the publisher.
