# Supplementary material for: Exercise-Based Renal Rehabilitation: A Bibliometric Analysis From 1969 to 2021
Source: Front Med (Lausanne). 2022 Mar 21;9:842919. doi: 10.3389/fmed.2022.842919 (PMC8977625; doi:10.3389/fmed.2022.842919)
Supplement: Supplementary file 1 [file Data_Sheet_1.pdf]

Supplementary File S1 Search Strategy for Web of Science Core Collection

TS=("exercise" OR "physical activity") AND TS=("predialysis" OR "pre-dialysis" OR "kidney disease" OR "renal disease" OR "kidney failure" OR "renal failure" OR "renal insufficiency" OR "kidney insufficiency" OR "hemodialysis" OR "haemodialysis" OR "hemofiltration" OR "haemofiltration" OR "hemodiafiltration" OR "haemodiafiltration" OR "dialysis" OR "renal transplantation" OR "kidney grafting" OR "kidney transplantation")

Supplementary File S2 Top ten citation analysis of reference on exercise-based renal rehabilitation

Rank	Reference	Citation
1	Johansen KL, Chertow GM, Ng AV, et al. Physical activity levels in patients on hemodialysis and healthy sedentary controls. Kidney Int. 2000;57(6):2564-2570. doi:10.1046/j.1523-1755.2000.00116.x	232
2	O'Hare AM, Tawney K, Bacchetti P, Johansen KL. Decreased survival among sedentary patients undergoing dialysis: results from the dialysis morbidity and mortality study wave 2. Am J Kidney Dis. 2003;41(2):447-454. doi:10.1053/ajkd.2003.50055	174
3	Kouidi E, Albani M, Natsis K, et al. The effects of exercise training on muscle atrophy in haemodialysis patients. Nephrol Dial Transplant. 1998;13(3):685-699. doi:10.1093/ndt/13.3.685	167
4	Heiwe S, Jacobson SH. Exercise training in adults with CKD: a systematic review and meta-analysis. Am J Kidney Dis. 2014;64(3):383-393. doi:10.1053/j.ajkd.2014.03.020	166
5	Painter P, Carlson L, Carey S, Paul SM, Myll J. Physical functioning and health-related quality-of-life changes with exercise training in hemodialysis patients. Am J Kidney Dis. 2000;35(3):482-492.	165

	doi:10.1016/s0272-6386(00)70202-2	
6	Heiwe S, Jacobson SH. Exercise training for adults with chronic kidney disease. Cochrane Database Syst Rev. 2011;(10):CD003236. Published 2011 Oct 5. doi:10.1002/14651858.CD003236.pub2	161
7	Johansen KL, Painter PL, Sakkas GK, Gordon P, Doyle J, Shubert T. Effects of resistance exercise training and nandrolone decanoate on body composition and muscle function among patients who receive hemodialysis: A randomized, controlled trial. J Am Soc Nephrol. 2006;17(8):2307-2314. doi:10.1681/ASN.2006010034	160
8	Cheema B, Abas H, Smith B, et al. Progressive exercise for anabolism in kidney disease (PEAK): a randomized, controlled trial of resistance training during hemodialysis. J Am Soc Nephrol. 2007;18(5):1594-1601. doi:10.1681/ASN.2006121329	156
9	Sietsema KE, Amato A, Adler SG, Brass EP. Exercise capacity as a predictor of survival among ambulatory patients with end-stage renal disease. Kidney Int. 2004;65(2):719-724. doi:10.1111/j.1523-1755.2004.00411.x	156
10	Konstantinidou E, Koukouvou G, Kouidi E, Deligiannis A, Tourkantonis A. Exercise training in patients with end-stage renal disease on hemodialysis: comparison of three rehabilitation programs. J Rehabil Med. 2002;34(1):40-45. doi:10.1080/165019702317242695	137
10	Johansen KL, Shubert T, Doyle J, Soher B, Sakkas GK, Kent-Braun JA. Muscle atrophy in patients receiving hemodialysis: effects on muscle strength, muscle quality, and physical function. Kidney Int. 2003;63(1):291-297. doi:10.1046/j.1523-1755.2003.00704.x	137
